# Combined hamstrings and peroneus longus tendon for undersized graft in anterior cruciate ligament reconstruction: A report of two adolescence female patients

**DOI:** 10.1016/j.ijscr.2020.09.136

**Published:** 2020-09-23

**Authors:** Andri M.T. Lubis, Dina Aprilya, Kanya Tania

**Affiliations:** Department of Orthopaedic and Traumatology, Faculty of Medicine Universitas Indonesia, Cipto Mangunkusumo Hospital, Indonesia

**Keywords:** ACL, anterior cruciate ligament, HT, hamstring tendon, PLT, peroneus longus tendon, SCARE, Surgical Case Report, IKDC, International Knee Documentation Committee, AOFAS, American Orthopedic Foot Ankle Score, ACL reconstruction, Female, Unqualified graft, Hamstring tendon, Peroneus longus tendon

## Abstract

**Introduction:**

Anterior cruciate ligament (ACL) rupture is a common injury in young and active population and reconstruction is the mainstay treatment to maintain stability of the knee. Hamstring tendon (HT) is the most popular graft for ACL reconstruction. In certain patient, particularly in women, the size of tendon graft to reconstruct the ACL is unpredictable. In the case of unqualified donor, we need a combined graft from another source to match the size requirement.

**Case presentation:**

We present two cases of ACL reconstruction using combination of HT and PLT graft in adolescent female. We evaluate knee functional score and ankle donor site morbidity. In the second case we also did second look for the ACL graft integrity arthroscopically. This study has been reported in line with the Surgical Case Report (SCARE) criteria.

**Discussion:**

One year after surgery, International Knee Documentation Committee (IKDC) score for both cases were improved and no ankle-donor site morbidity with good motoric power and 100% American Orthopedic Foot Ankle Score (AOFAS).

**Conclusion:**

Additional peroneus longus tendon can be considered as an alternative in undersized hamstring tendon graft without significant donor site morbidity.

## Highlights

• Unpredictable graft size for anterior cruciate ligament reconstruction.
• Plan for graft augmentation is of great importance.
• Combined graft from hamstring and peroneus longus tendon for undersized graft.

## 1 Introduction

The variability of graft choices in ACL reconstruction urges clinicians to individualize the choice based on each patient by considering graft availability, patient’s preference and characteristics such as age, sex, anthropometric status and activities [1]. Hamstring tendon (HT) is the most commonly used as it has lesser morbidity regarding to knee extension deficit and kneeling pain [2,3]. However, HT graft has an unpredictable size and thus can be problematic in smaller tendon size. Another source of graft is peroneus longus tendon (PLT) graft which still in debates due to the donor site morbidity despite of its strength and stiffness, its use as augmentation graft was a good option due to the easy access and lesser infection risk [[4], [5], [6], [7]].

In this report, we combined HT and PLT graft in two adolescence female patients with similar characteristic and evaluated the knee and ankle stability parameters. Written consent was obtained from parents for publication of this case report and accompanying images. The authors declare no conflicts of interest. This work has been reported in line with the SCARE criteria [8].

## 2 Case presentation

Two patients presented to our center with knee instability following a twisting injury. The first patient was 15-year-old female with 50 kg of weight and 150 cm in height with a confirmed ACL rupture on Magnetic Resonance Imaging (MRI). The second patient was 16-year-old female with 55 kg weight and 155 cm height with a confirmed ACL rupture and meniscus injury on MRI. We performed ACL reconstruction only in the first patient and ACL reconstruction with meniscus repair in the second patient.

### 2.1 Surgical techniques

#### 2.1.1 Hamstring tendon harvesting

**Fig. 1:**
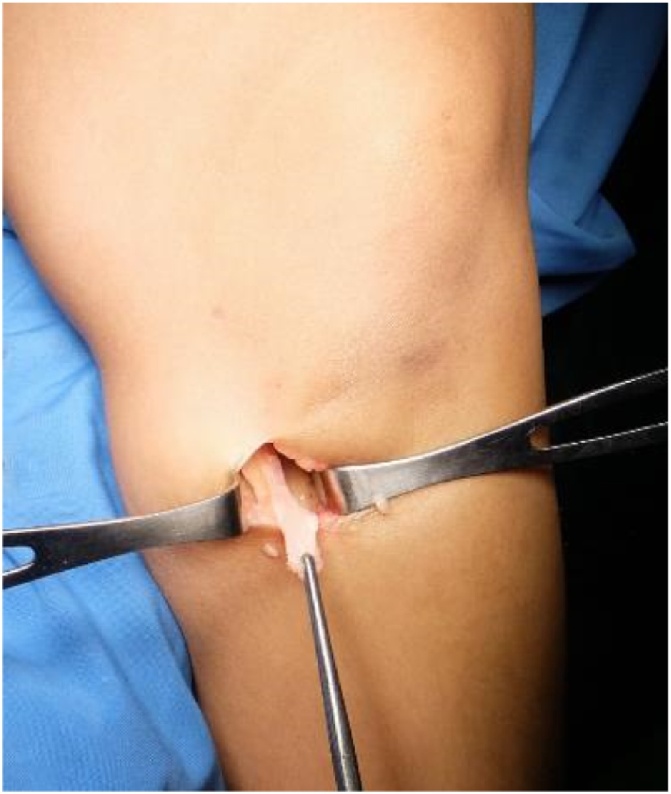
Hamstring Tendon Graft harvesting.

After drawing the osseous landmark at patella and tibial tubercle, an oblique 2 cm incision was made along the pes anserinus between tibial tubercle and posteromedial border of tibia. Gracilis and semitendinosus tendons were identified and harvested (Fig. 1). The distal end of the semitendinosus tendon and gracilis tendon were lifted up with scissors and grasped with a Kocher. The conjoined tendon between tendon distal ends was divided and released. By advancing the tendon stripper, tendons were detached from the muscle proximally.

#### 2.1.2 Peroneus longus tendon harvesting

**Fig. 2:**
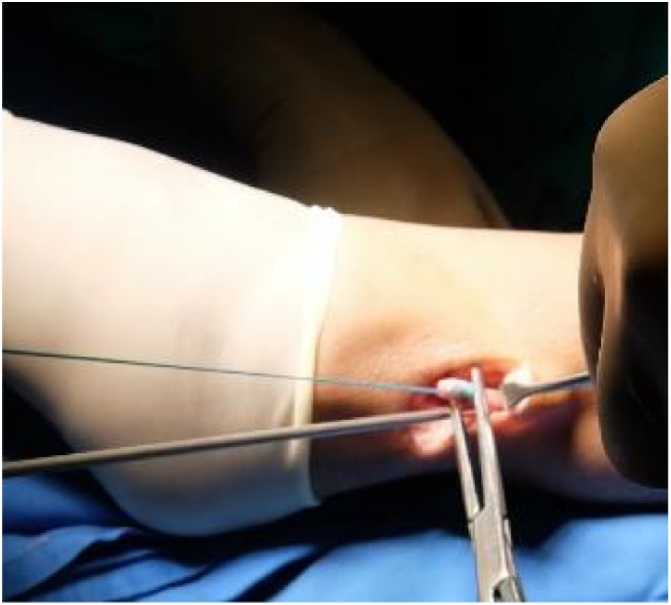
Peroneus longus tendon graft harvesting.

PLT was harvested through an incision along the posterior border of the distal fibula, above the superior peroneal retinaculum. The tendon was exposed on its posterolateral surface. The distal end was cut and held with suture and the proximal end was released by tendon stripper (Fig. 2).

In the first patient, HT graft by combining gracillis and semitendinosus tendon was only 5 mm in diameter with 13 cm length after quadrupled (Fig. 1). This 5 mm diameter graft was too small to be a good graft. To salvage this unqualified graft, we used PLT graft. The 6 mm diameter and 11 cm length-harvested PLT was added to the unqualified HS graft and made an 8 mm × 11 cm six-strand graft.

Meniscus was repaired in the second patient and ACL reconstruction was planned with PLT graft considering the activity level and the similarity of body characteristic as the previous patient. We harvest the PLT with the same manner as previous patient. The tendon was surprisingly insufficient 6 mm × 10 cm (after doubled). We decided to take the HT as augmentation and a final 7.5 mm × 10 cm six strand graft was obtained.

In both patients, autografts were fixed to the femoral tunnel with Endo-button (Smith & Nephew) and to the tibial tunnel with interference screw (Smith & Nephew).

Post operatively, the first patient had the knee immobilized in 0° extension in a week and gradually increase until 90° flexion in the second week. Afterwards the brace was set in flexible range so that patient could start the active and passive range of motion exercise. Weight bearing was increased as tolerated. The second patient had a combined ACL reconstruction and meniscus repair rehabilitation protocol. In both patients, gentle ankle stretch was encouraged since the first post-operation day by using a resistance band. Proprioceptive exercises were performed 3 months after surgery which were gradually increased in intensity until another 3 months.

### 2.2 Evaluation

Pain and stability at knee and ankle joint subjectively and objectively evaluated at one-year post operatively. We used the visual analogue scale (VAS) to measure pain before and after surgery. Knee and ankle stability were evaluated using the International Knee Documentation Committee (IKDC) 2000 Standard Evaluation Form and American Orthopedic Foot and Ankle Society (AOFAS) Ankle-Hindfoot Score respectively. The power of foot eversion and first ray plantar flexion was examined using Medical Research Council (MRC) scale for muscle strength and compared to the normal sides on contralateral ankles. Other donor-site morbidities at ankle region were also recorded such as numbness or proximal stump irritation. In the second patient ACL graft was re-evaluated on the second look 8 months after the reconstruction.

## 3 Results

### 3.1 Knee pain and IKDC subjective knee evaluation

In the first patient, knee pain was VAS 3–4 before surgery and VAS 0 on one year follow up. IKDC Score 100% at one year follow up. In the second case, the knee pain was VAS 3 before surgery, and now only experienced mild discomfort. IKDC score was 95.4% at one year follow up.

### 3.2 Knee stability test

Both of patient knees were unstable pre-operatively as observed by the positive measurement on anterior drawer test, pivot shift test and Lachman test (+3). Both patients had negative Lachman and anterior drawer test in one year follow up.

### 3.3 Ankle morbidity

**Fig. 3:**
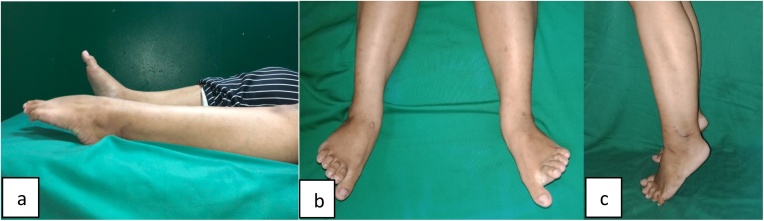
Full ROM (a) plantar flexion and (b) eversion; (c) standing on tip toe at one year follow up.

In both patients, no complaint regarding the ankle with good motoric power (scale 5 for both ankle eversion and first ray plantar flexion) and no limitation of ankle range of motion (Fig. 3). The 100% AOFAS score were observed in both patients at one-year follow up.

### 3.4 Second-look arthroscopy

**Fig. 4:**
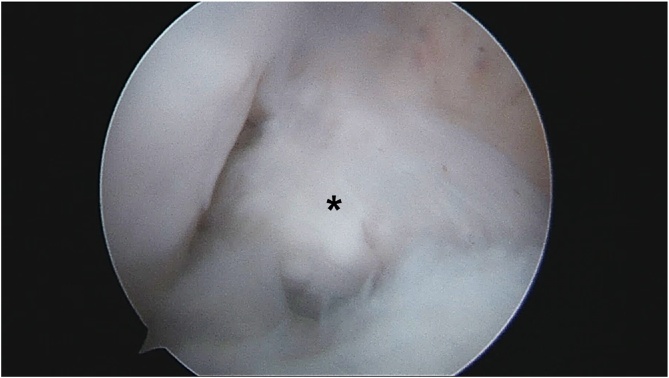
ACL graft (Asterix) observed via the arthroscopic anterolateral viewing portal on second-look arthroscopy.

The second patient agreed to undergo second-look arthroscopy 8-months after ACL reconstruction. Graft was taut on probing, completely covered by synovium, and no tear was observed (Fig. 4).

## 4 Discussion

Besides females have a more common ACL injury than males, they also have some special considerations regarding to the reconstruction [[9], [10], [11]]. Female HTs are shorter in length and smaller in diameter compared to male patient. The tensile strength of the hamstring tendon is also weaker in female as laxity is more common which is also plays as contributing factor for the injury itself. Some studies revealed moderate correlation of the HT graft size and anthropometric parameters, which can be valuable for patient counseling and alternative graft source planning. Both of our patients had similar anthropometry profile which actually not at risk for an inadequate graft size [3,4,12].

The common problem regarding the reconstruction in female is usually the graft size mismatch [12,13]. To ensure appropriate graft size and biomechanical function, it has been recommended to use grafts at least 7–8 mm in diameter to replicate the native ACL diameter [2,14]. In this report, our patients were both adolescence female with similar characteristic. In the first patient, we preferred HT despite of the possibility of undersized graft in Asian female with such anthropometric profile because the lesser risk of anterior knee pain. Graft salvage method has been prepared if the HT were undersized such as PLT or opposite HT. Based on the previous patient with the similarity of characteristic and previous activity, we choose primary PLT graft for ACL reconstruction. However, due to the undersized PLT, we harvested HT for augmentation.

PLT is one of the important supportive structure to provide stability of the foot and ankle. Chayanin et al. [5] study not recommend the use of PLT graft as the first option for the ACL reconstruction but this tendon can serve as an alternative donor in case of the multi-directional instability which needs several donor tendons in the reconstruction. In contrast, other studies evaluated the ACL reconstruction using PLT autograft and the study has shown that most patients rated as normal or nearly normal IKDC score with no effect on gait parameters, ankle joint dysfunction or difficulty in sports activities [[4], [5], [6], [7],^15^].

This study used PLT as augmentation graft. IKDC scores were improved to normal. The AOFAS score were excellent, similar with previous result by Chayanin et al. [5] and Rhatomy et al. [16] which investigated ankle functional outcome after PLT harvesting. Knee stability parameters in our patients were also improved. Furthermore, there were no ankle pain, numbness and ankle-foot motion restriction in post-operative follow-up. Subjectively, the muscle strength of ankle plantar flexion and foot eversion were accessed and result in normal muscle strength. Patient also can perform tip toe walking by means that there was no limitation of the ankle and foot function post operatively. This finding supported the study by Kermoglu et al. [7] which found no dysfunction or any difficulty in sports activities at their follow up with improved knee stability profile. Shi et al. [17] also showed no differences between preoperative and postoperative ankle strength and range of motion.

Limitations of this study include are small number of participants and the need of quantified data for the ankle muscle power with longer period of evaluation regarding the long-term ankle morbidity outcome.

## 5 Conclusion

Uncertainty in predicting graft size urged the preoperative planning to prepare graft augmentation. Combining hamstrings tendon with peroneus longus tendon could be an alternative for undersized graft without a significant ankle-donor site morbidity.

## Declaration of Competing Interest

The authors report no declarations of interest.

## Funding

The authors report no external source of funding during the writing of this article.

## Ethical approval

Ethical approval was not required in the treatment of the patient in this report.

## Consent

Written informed consent was obtained from the parents for publication of this case report and accompanying images. A copy of the written consent is available for review by the Editor-in-Chief of this journal on request.

## Author contribution

Dina Aprilya and Kanya Tania contribute to the study concept or design, data collection and writing the paper.

Andri MT Lubis contributes in the study concept or design, data collection, analysis and interpretation, oversight and leadership responsibility for the research activity planning and execution, including mentorship external to the core team.

## Guarantor

Andri MT Lubis is the sole guarantor of this submitted article.

## Provenance and peer review

Not commissioned, externally peer-reviewed.

## References

[1] Bonasia D.E., Amendola A., Menetrey J. (2012). The traumatic knee: graft choice in ACL reconstruction. The Knee Joint Surgical Techniques and Strategies.

[2] Maeda A., Shino K., Horibe S., Nakata K., Buccafusca G. (1996). Anterior cruciate ligament reconstruction with multistranded autogenous semitendinosus tendon. AOSSM.

[3] Siebold R., Webster K.E., Feller J.A., Sutherland A.G., Elliott J. (2006). Anterior cruciate ligament reconstruction in females: a comparison of hamstring tendon and patellar tendon autografts. Knee Surg. Sports Traumatol. Arthrosc..

[4] Liu C.T., Lu Y.C., Huang C.H. (2015). Half‑peroneus‑longus‑tendon graft augmentation for unqualifed hamstring tendon graft of anterior cruciate ligament reconstruction. J. Orthop. Sci..

[5] Angthong C., Chernchujit B., Apivatgaroon A., Chaijenkit K., Nualon P., Suchao K. (2015). The anterior cruciate ligament reconstruction with the peroneus longus tendon: a biomechanical and clinical evaluation of the donor ankle morbidity. J. Med. Assoc. Thai..

[6] Nazem K., Barzegar M.R., Hosseini A., Karimi M.T. (2015). Can we use peroneus longus in addition to hamstring tendons for anterior cruciate ligament reconstruction?. Adv. Biomed. Res..

[7] Kerimoglu S., Aynaci O., Saracoglu M., Aydin H., Turhan A.U. (2008). Anterior cruciate ligament reconstruction with the peroneus longus tendon. Acta Orthop. Traumatol. Turc..

[8] Agha R.A., Borrelli M.R., Farwana R., Koshy K., Fowler A., Orgill D.P., For the SCARE Group (2018). The SCARE 2018 statement: updating consensus surgical CAse REport (SCARE) guidelines. Int. J. Surg..

[9] Marrale J., Morissey M.C., Haddad F.S. (2007). A Literature review of autograft and allograft anterior cruciate ligament reconstruction. Knee Surg. Sports Traumatol. Arthrose..

[10] Shino K., Mae T., Tachibana Y. (2005). Anatomic ACL reconstruction: rectangular tunnel/ bone-patellar tendon-bone or triple bundle/semitendinosus tendon grafting. J. Orthop Sci..

[11] Prodromos C.C., Han Y., Rogowski J., Joyce B., Shi K. (2007). A meta-analysis of the incidence of anterior cruciate ligament tears as a function of gender, sport, and a knee injury-reduction regimen. Arthroscopy.

[12] Sutton K.M., Bullock J.M. (2013). Anterior cruciate ligament rupture: differences between males and females. J. Am. Acad. Orthop. Surg..

[13] Insall J.N., Torzilli P.A., Greenberg R.L., Hood R.W., Pavlov H. (1984). Measurement of anterior-posterior motion of the knee in injured patients using a biomechanical stress technique. J. Bone Joint Surg. Am..

[14] Frank R.M., Hamamoto J.T., Bernardoni E., Cvetanovich G., Bach B.R., Verma N.N. (2017). ACL reconstruction basics: quadruple (4-Strand) hamstring autograft harvest. Arthrosc. Tech..

[15] Shaerf D., Pastides P., Sarraf K., Willis-owen C. (2014). Anterior cruciate ligament reconstruction best practice: a review graft choice. World J. Orthop..

[16] Rhatomy S., Asikin A.I.Z., Wardani A.E., Rukmoyo T., Lumban-Gaol I., Budhiparama N.C. (2019). Peroneus longus autograft can be recommended as a superior graft to hamstring tendon in single-bundle ACL reconstruction. Knee Surg. Sports Traumatol. Arthrosc..

[17] Shi F.D., Hess D.E., Zuo J.Z. (2019). Peroneus longus tendon autograft is a safe and effective alternative for anterior cruciate ligament reconstruction. J. Knee Surg..

